# Informing spatial conservation prioritization with species’ traits

**DOI:** 10.1111/cobi.70199

**Published:** 2025-12-13

**Authors:** Liam A. Trethowan, Laura Jennings, Haerul Arifin, Renata Borosova, Gemma L. C. Bramley, Marie Briggs, Osanna Chu, Ruth P. Clark, Sally Dawson, Kiran L. Dhanjal‐Adams, Yance de Freitas, James Hartup, Edward G. E. Heatubun, Cecilia Lee‐Grant, Laurensia V. Mapandin, Jonni Marwa, Alison Moore, Agustinus Murdjoko, Carmen Puglisi, André Schuiteman, Dave J. I. Seaman, Nurhaidah Sinaga, Matthew J. Struebig, Anna Trias Blasi, Timothy Utteridge, Maria Voigt, Jimmy F. Wanma, Charlie D. Heatubun

**Affiliations:** ^1^ Herbarium Kew London UK; ^2^ Badan Riset Inovasi Daerah Manokwari Indonesia; ^3^ Royal Botanic Gardens Edinburgh Edinburgh UK; ^4^ School of Geography, Earth and Environmental Sciences University of Birmingham Birmingham UK; ^5^ School of Environmental Sciences University of Liverpool Liverpool UK; ^6^ Department of Biology Universitas Gadjah Mada Yogyakarta Indonesia; ^7^ Department of Life Sciences Imperial College London London UK; ^8^ Fakultas Kehutanan Universitas Papua Manokwari Indonesia; ^9^ Forests and Climate Change Programme (FORCLIME) Deutsche Gesellschaft fur Internationale Zusammenarbeit (GIZ) Sorong Indonesia; ^10^ Missouri Botanical Garden St. Louis Missouri USA; ^11^ Durrell Institute of Conservation and Ecology University of Kent Canterbury UK; ^12^ Botanical Research Singapore Botanic Gardens Singapore Singapore; ^13^ The Nature Conservancy Berlin Germany; ^14^ Faculty of Mathematics and Natural Sciences Universitas Indonesia Depok Indonesia

**Keywords:** plants, systematic conservation planning, taxonomic descriptions, tropical forest, West Papua, bosque tropical, descripciones taxonómicas, Papúa occidental, planeación de la conservación sistemática, plantas, 植物, 系统性保护规划, 分类学描述, 热带森林, 西巴布亚

## Abstract

New Guinea, the most botanically diverse island on the planet, is the location for one of the boldest conservation initiatives. The Manokwari Declaration aims to achieve 70% conservation designation for the Bird's Head Peninsula. This is 40% higher than the 2022 Global Biodiversity Framework target. However, there is a lack of species occurrence data to support evidence of where biodiversity can be best protected. To address this, we integrated plant trait data from taxonomic descriptions in species occurrence models that can inform conservation planning. Inclusion of traits improved the performance of co‐occurrence models of ∼800 plant species across the 100,000‐km^2^ landscape. Traits generally improved model performance, but not all traits contributed equally (e.g., leaf size and red flower color most improved accuracy of occurrence prediction). Likewise, trait‐parameterized models tended to be most useful with rare species occurrence prediction, but this was inconsistent among traits. Under 70% protection, three‐quarters of the areas selected conserved trait diversity. Critically, trait diversity also increased the chances that areas at high risk of deforestation were selected as conservation priorities. Overall, we found that plant species’ traits, often key parameters of ecosystem function and resilience, improved spatial conservation planning.

## Abstract (es)

Información sobre los rasgos de las especies para la priorización de la conservación espacial

**Resumen:**

Nueva Guinea, la isla con mayor diversidad botánica del planeta, es el escenario de una de las iniciativas de conservación más audaces. La Declaración de Manokwari tiene como objetivo lograr que el 70% de la península de Bird's Head sea designada como zona de conservación. Esto supone un 40% más que el objetivo del Marco Mundial para la Diversidad Biológica de 2022. Sin embargo, faltan datos sobre la presencia de especies que respalden las pruebas sobre dónde se puede proteger mejor la biodiversidad. Para solucionar este problema, integramos datos sobre las características de las plantas procedentes de descripciones taxonómicas en modelos de presencia de especies que pueden servir de base para la planificación de la conservación. La inclusión de las características mejoró el rendimiento de los modelos de coocurrencia de unas 800 especies de plantas en un paisaje de 100, 000 km^2^. En general, las características mejoraron el rendimiento del modelo, pero no todas contribuyeron por igual (por ejemplo, el tamaño de las hojas y el color rojo de las flores mejoraron en mayor medida la precisión de la predicción de la presencia). Del mismo modo, los modelos parametrizados por rasgos tendían a ser más útiles para la predicción de la presencia de especies raras, pero esto no era uniforme entre todos los rasgos. Con una protección inferior al 70%, tres cuartas partes de las áreas seleccionadas conservaban la diversidad de rasgos. Es importante destacar que la diversidad de rasgos también aumentaba las posibilidades de que las áreas con alto riesgo de deforestación fueran seleccionadas como prioridades de conservación. En general, descubrimos que los rasgos de las especies vegetales, que a menudo son parámetros clave de la función y la resiliencia de los ecosistemas, mejoraban la planificación de la conservación espacial.

## 利用物种性状指导空间优先保护

**【摘要】** 新几内亚作为地球上植物多样性最丰富的岛屿, 正在实施一项最具雄心的保护计划。马诺夸里宣言旨在将鸟头半岛(Bird's Head Peninsula)的保护区比例提升至70%, 这一目标比《2022年全球生物多样性框架》目标高出40%。然而, 目前尚缺乏物种分布数据, 来指导生物多样性保护最适宜的选址。为此, 我们将植物分类学描述中的性状数据整合到物种分布模型中, 从而为保护规划提供依据。在10万平方公里的景观中, 纳入性状数据提升了约800种植物的共现模型的预测表现。性状数据普遍能够提升模型表现, 但贡献程度存在差异, 例如, 叶片大小与红色花朵特征对分布预测准确性的提升最为显著。类似地, 性状参数化模型在稀有物种分布预测中效果最佳, 但不同性状间的表现存在差异。在70%的保护比例下, 所选区域中有四分之三保护了性状多样性。关键的是, 纳入性状多样性还提高了高砍伐风险区域被选为优先保护区的概率。总体而言, 我们发现植物物种的性状(通常是生态系统功能与恢复力的关键参数)有助于优化空间保护规划。**【翻译:胡怡思;审校:聂永刚】**

## INTRODUCTION

Species diversity is highest in the tropics (Mittelbach et al., 2007; Raven et al., 2020), where prioritization of conservation areas is especially critical and often requires a balance between species retention and sustainable development (Barrett et al., 2001; Rosa et al., 2017). This is particularly acute on the Bird's Head Peninsula in New Guinea, the world's most floristically diverse island (Cámara‐Leret et al., 2020). The botanical megadiversity of this region faces threats from expansion of agriculture, mining, and selective logging, which have led to ∼235,000 ha of forest loss from 2001 to 2019 (Austin et al., 2019; Gaveau et al., 2021; Parker et al., 2024). However, one of the most ambitious conservation goals on the planet has been set there by the Manokwari Declaration (Cámara‐Leret, Schuiteman, et al., 2019). The declaration designates 70% of the Bird's Head Peninsula for conservation, roughly 70,000 km^2^, and seeks to balance economic and infrastructure development with cultural and environmental protection, the rights of Indigenous peoples, and carbon storage (Cámara‐Leret, Schuiteman, et al., 2019; Sloan et al., 2019). Conservation plans are therefore required to promote sustainable development in the region (Parsch et al., 2022). If plans devised to meet the declaration's goal cannot come to fruition, the region would implement the Convention on Biological Diversity's (CBD) less ambitious target to conserve 30% of land area by 2030 (CBD, 2022; Cobos et al., 2023; Harris et al., 2024).

However, most tropical species are rare and do not have enough occurrence data for computationally intensive, data‐heavy, spatial conservation prioritization assessments (Feeley & Silman, 2011; Pusparini et al., 2023; Sibarani et al., 2019; Silvestro et al., 2022). The scarcity of data limits the robustness of species distribution models that often underpin spatial planning (Hanson et al., 2025; Moilanen, 2007). This is especially pronounced for tropical plants, most species of which have few occurrence records (Enquist et al., 2019; Hughes et al., 2021; Jeliazkov et al., 2022) and associated data, including trait information (Cornwell et al., 2019; Maitner et al., 2023). Given the critical role of plant species in ecosystem services and functions (Cámara‐Leret, Raes, et al., 2019; Gross et al., 2017; Le Bagousse‐Pinguet et al., 2019; Liang et al., 2016), addressing the shortfall of botanical data will allow effective conservation planning to limit deforestation‐driven loss of biodiversity and carbon stocks.

The physiological traits of plants result from and affect the environment, mutualism, competition, herbivory, and pathogens (e.g., Westoby et al., 2023). This critical role in ecological relationships has made traits central to models of biodiversity, ecosystem function, and services (Weiskopf et al., 2022). Species’ traits can be used to improve species distribution modeling and are another facet of biodiversity to consider in spatial conservation planning (Butt & Gallagher, 2018; Cadotte et al., 2011; Dudley et al., 2019; Gallagher et al., 2021). The interaction between plant species’ traits and the environment shape where species occur (e.g., Alzate & Onstein, 2022; Velásquez‐Puentes et al., 2023). For example, small leaves, flowers, and fruit generally mean a species tolerates drought through traits that reduce water loss (Estrada et al., 2015, 2016; Trethowan et al., 2023; Trethowan, Jennings, et al., 2024). Similarly, plants living at high elevations are adapted to cold, although in this case flower size can increase due to greater relative abundance of large pollinators (Fajardo et al., 2024; Herrera, 2005; Trethowan, Jennings, et al., 2024). Given the lack of occurrence data to model plant distributions, traits can be used to improve assemblage predictions, and trait information can complement the use of species taxonomic diversity to predict key ecosystem functions and services (Ross et al., 2021; Santini et al., 2016; Sellan et al., 2024). Carbon storage, the most used measure of ecosystem function, depends on traits and trait diversity in a community (Finegan et al., 2015). Traits predict species’ responses to stressors (Comte et al., 2024; Siefert & Laughlin, 2023). Considering trait diversity in prioritization frameworks, therefore, gives a better chance to retain valuable ecosystems, carbon stocks, and identify areas most at risk under future conditions (Gallagher et al., 2021; Harris et al., 2024; Pollock et al., 2020).

We used plant species trait data and the CBD 30% and the Manokwari Declaration 70% protection targets in conservation planning models to test whether trait inclusion in distribution models and spatial prioritization improves protection. We considered 4 key questions regarding the best use of traits: which traits are most valuable for species co‐occurrence model improvement, are trait‐parameterized models beneficial for rare species occurrence prediction, does trait diversity affect conservation prioritization scores, and does trait diversity aid prioritization for conservation of areas important for storage of aboveground carbon and areas at risk of deforestation?

Answering these questions can elucidate the importance of traits in species distribution modeling prior to and during conservation prioritization. We also compared prioritizations for biodiversity only with those in which biodiversity, carbon, and deforestation risk were combined.

## METHODS

### Traits from taxonomic descriptions

**FIGURE 1:**
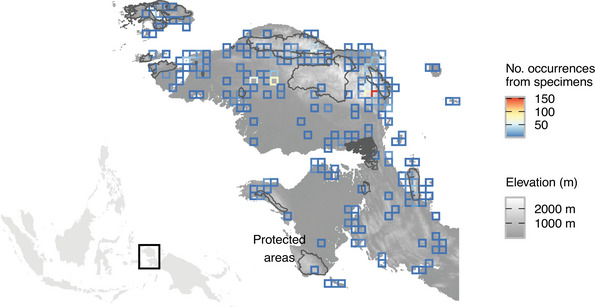
Elevation, protected areas, and species occurrence density in the Bird's Head landscape of Indonesian New Guinea. Occurrence data are based on herbarium specimen georeferences in 11‐km^2^ planning units. Inset shows study location in archipelagic southeast Asia.

We built a species list with 2203 expert‐verified and georeferenced herbarium specimen occurrence records (Figure 1) from the Bird's Head Peninsula sourced from complete Flora Malesiana accounts (e.g., Bramley et al., 2019; de Wilde, 2000) or from a large‐scale collation of occurrence data (L.J. unpublished data; M.B. unpublished data). We gathered traits for these 836 species, from 246 taxonomic publications, compiling data on plant, leaf and fruit size, plant habit variables (tree, shrub, climbing, herb, and epiphyte), and flower color. We used the largest recorded measurements for plant size and for the 2 leaf size categories, length and width (Trethowan et al., 2022). For fruit size, we used the largest recorded measurement of either length or width (Trethowan et al., 2023). Flower size was the largest recorded measurement of calyx, corolla, or stamen length (Trethowan, Jennings, et al., 2024). Flower color was split into 7 dichotomous variables: red, yellow, white, purple, pink, orange, and brown (Trethowan, Jennings, et al., 2024). All continuous data were transformed to the log and scaled as *z* scores prior to trait imputation, co‐occurrence modeling, and trait diversity calculation.

Thirty‐eight, 291, 35, 56, 235, and 496 species lacked data on habitat, size, leaf length, leaf width, flower size, and fruit size, respectively. Missing data were far below the 70% threshold where diversity indices are expected to be affected after gap filling (Stewart et al., 2023). To fill continuous and categorical trait data gaps, we ran random forest models that incorporated the first 10 selected phylogenetic eigenvectors to aid imputation accuracy (Carmona et al., 2024; Penone et al., 2014; Stekhoven & Bühlmann, 2012). We repeated imputation of continuous traits with a maximum likelihood estimation informed by phylogenetic covariance (Bruggeman et al., 2009; Goolsby et al., 2017). Imputation models were iterated 15 times and average values for species were calculated for each continuous trait, and the most frequently predicted value for categorical traits was selected.

The phylogeny used for imputation was built for all species in the dataset, and we assumed a Brownian motion model of evolution (Li et al., 2020). To construct the phylogeny, dated phylogenetic data were acquired from published phylogenies (Jin & Qian, 2019). When only genera were present in the backbone phylogeny, species were added as a polytomy at the root node (i.e., most recent common ancestor) of the genus. Genera not present in the backbone phylogeny were bound to the halfway point between the family root node and basal node. When the family branch length was longer than two‐thirds from the family root node to the tip, the genus was bound to the upper third of the entire family branch length (Jin & Qian, 2019; Qian & Jin, 2016). This followed methods implemented previously with different software (i.e., Phylomatic and BLADJ) (Webb et al., 2008).

### Environmental parameters

We used principal component (PC) analyses to select 3 noncovarying environmental parameters for our models of species co‐occurrence (Cámara‐Leret, Raes, et al., 2019). We included 13 scaled and centered variables: elevation from the Shuttle Radar Topography Mission (Farr et al., 2007), annual mean temperature, mean diurnal range, isothermality, temperature seasonality, temperature annual range, annual precipitation and precipitation seasonality from WorldClim (Fick & Hijmans, 2017), bulk density of fine earth fraction, volumetric fraction of coarse fragments, pH, sand in fine earth, and soil organic carbon from soil grids (Hengl et al., 2017). Environmental data were averaged (mean) for 11 × 11‐km grid cells to match area of planning units used in downstream analyses. Following Cámara‐Leret, Raes, et al. (2019), we selected the 3 variables with greatest correlation with each of the first 3 PC axes (PC1 and mean annual temperature = 0.47, PC2 and temperature seasonality = 0.51, PC3 and soil organic carbon = 0.55). These 3 PC axes explained 64% (first axis 30%, second 19%, third 15%) of the total variation in environmental parameters.

### Species co‐occurrence model

**FIGURE 2:**
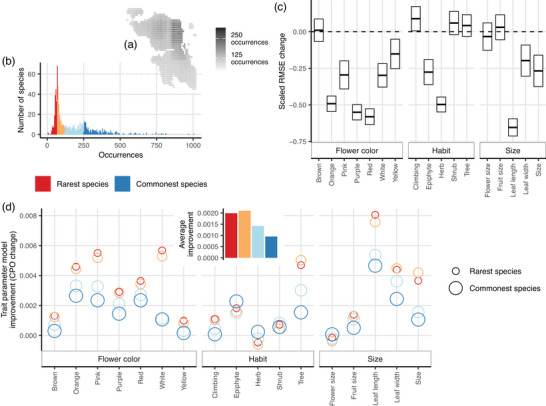
On the Bird's Head Peninsula, New Guinea, for plant species, the (a) mean number of species’ occurrences in planning units across 5 distribution datasets used, (b) mean number of occurrences for species across 5 distribution datasets separated into rarity quartiles, (c) Bayesian phylogenetic co‐occurrence model improvement when trait and trait × environment interaction are included (lines in bars, mean; bar ends, standard error; RMSE, root mean square error from models including trait parameters; model improvement, null 10‐fold cross‐validation—RMSE), and (d) model improvement as species median conditional predictive ordinate (CPO) difference between trait and nontrait parameterized models for rarity quartiles (inset, same data but median values for rarity quartiles across all models).

We modeled co‐occurrence of plant species across the Bird's Head Peninsula of Indonesian New Guinea (Figure 2a) with integrated nested Laplace approximations (Martins et al., 2013). We used the following Bayesian Gaussian phylogenetic mixed‐effects joint species distribution model:

$$Y_{i}=\alpha +\beta \gamma +\beta \delta +\beta \omega +a_{\mathrm{spp}\left[i\right]}+b_{\mathrm{spp}\left[i\right]}+c_{\mathrm{pu}\left[i\right]}+d_{\mathrm{pu}\left[i\right]},$$

$$a∼\mathrm{Gaussian}\left(0,\sigma_{a}^{2}I_{n}\right),$$

$$b∼\mathrm{Gaussian}\left(0,\sigma_{b}^{2}\Sigma_{\mathrm{spp}}\right),$$

$$c∼\mathrm{Gaussian}\left(0,\sigma_{c}^{2}I_{m}\right),$$

(1) $$d∼\mathrm{Gaussian}\left(0,\sigma_{d}^{2}\Sigma_{m}\right),$$

where Greek letters are fixed effects; other letters are mixed effects (Gelman & Hill, 2006); $Y_{i}$ is the probability of observations i of presence n in 1016 planning units m that are approximately 11 × 11‐km grid cells in the Bird's Head Peninsula (Figure 2a); β is trait; γ is mean annual temperature; δ is temperature seasonality; ω is soil organic carbon; α is the estimated species average presence in each planning unit m; $a_{\mathrm{spp}[i]}$ is a random effect of species identity for which covariance was drawn from a Gaussian distribution with mean zero and $\sigma^{2}$ variance; $b_{\mathrm{spp}[i]}$ is random effect of species identity for which covariance in species effects was based on $\sigma_{b}^{2}\Sigma_{\mathrm{spp}}$; and $\Sigma_{\mathrm{spp}}$ is the matrix of phylogenetic branch length distances between species. This calculation allowed us to account for species not being independent samples because they share evolutionary history, which influences response to the environment and species’ traits (Gervasi & Schiestl, 2017; Li & Ives, 2017; Li et al., 2019; Vasconcelos et al., 2019; Westoby et al., 2023). The phylogenetic distance matrix was calculated from the same phylogeny used for trait imputation. Planning unit included 2 random effects: $c_{\mathrm{pu}[i]}$, drawn from a Gaussian distribution with mean zero and variance $\sigma^{2}$, and $d_{\mathrm{pu}[i]}$, for which covariance was based on $\sigma_{b}^{2}\Sigma_{m}$. The spatial covariance matrix consisted of the scaled exponential distances between unit centroids (French, 2018). In tandem, these random effects accounted for unmeasured environmental variation in units and the greater chance that closer units will share species or environmental characteristics (Dray et al., 2012). The probability of species presence was modeled as a function of β and its interactions with γ, δ, and ω.

We ran separate models for each β with either a binomial predictor for flower colors and habits or a Gaussian predictor for flower, fruit, and plant size. A Gaussian probability estimator was used to allow computation of occurrence probability for 836 species across 1016 planning units (Ives, 2018). For downstream analyses, Gaussian output was rescaled from 0 to 1 to give relative occurrence probability of species, and these values were averaged for planning units for each species per model run for each trait (Ives, 2018; Li et al., 2020).

We repeated models for 5 sources of occurrence data. We first used species presence from specimen data. This dataset was prone to type 2 errors caused by low collection effort (Cámara‐Leret et al., 2020; Feeley & Silman, 2011). We addressed this with 3 approaches, a 50‐km and a 150‐km radius of presence around points that addressed localized type 2 errors and by designating species as present in the bounding box of their specimen occurrence points (i.e., used species minimum and maximum longitude and latitude). Although these 3 approaches reduce type 2 errors, they can increase type 1 errors by falsely assigning species as present, particularly at erroneous elevations in the spatial range of a species. To address this, we reran the models a fifth time with species designated as present at all elevations in the elevation range recorded in species’ taxonomic descriptions. This resulted in a species mean number of presences in planning units across these datasets of 178 (Appendix S1). Occurrence probabilities from model runs for each trait and presence‐background dataset (Appendix S2) were averaged for each species in each planning unit. This approach was aimed at balancing the strengths and weaknesses of presence‐background datasets so overall results would reflect the environment occupied by species and overcome the dearth of occurrence points in the tropics (Graham et al., 2008; Jeliazkov et al., 2022; Muscatello et al., 2021; Oliveira et al., 2024).

To determine how traits improve the co‐occurrence modeling framework, we ran null models with both trait parameters and the environment–trait interaction parameter removed. We primarily quantified model improvement with root mean square errors (RMSE) from 10‐fold cross‐validation (Moraga, 2019; Wilkinson et al., 2021) (Appendix S3 & S4). These were calculated separately for null and trait models for all 3 distribution datasets. To allow comparison between distribution datasets, we scaled each set of RMSE scores separately between zero and one. We then subtracted null RMSEs from trait model values, resulting in relative measures of model improvement per trait. We also calculated conditional predictive ordinate (CPO) values (i.e., probability of observing an occurrence when the model is fit without the occurrence in question) (Gómez‐Rubio, 2020). Higher CPO values indicate greater prediction accuracy (Held et al., 2010). To identify whether traits altered model performance uniformly among common and rare species, we compared median CPO values between rarity quartiles. Rarity quartiles were assigned from the mean number of occurrences across distribution datasets (Figure 2b; Appendix S1). We compared quartiles across all models and independently per trait.

### Prioritization feature calculation

For our prioritization, we used the prioritizr algorithm (Hanson et al., 2024, 2025) and the HiGHS solver (Huangfu & Hall, 2018) to maximize coverage of our conservation features, balanced against the cost of land, when designating potential protected areas. Our conservation features were species and trait diversity metrics that follow the Hill number framework (Chao, Chiu, et al., 2014). Hill numbers obey the replication principle in that when 2 assemblages have zero species in common and identical relative abundance distributions, when summed their diversity is twice as high. This is not the case for classic abundance‐weighted diversity measures (e.g., Shannon entropy and Gini–Simpson) (Chao, Chiu, et al., 2014). Hill number increases are therefore easier to interpret and subsequently useful for conservation prioritization (Jost et al., 2010).

For all diversity metric calculations, we used a relative abundance input. This was the summed presence from 20 random draws, with species relative occurrence probabilities in planning units taken from co‐occurrence models. High occurrence probabilities are more likely to be selected as present per draw. We expected our planning units to have underestimated species sampling given they were in the undersampled tropics (Cazzolla Gatti et al., 2022; Feeley & Silman, 2011). We therefore extrapolated Hill alpha diversity metrics to give 98.5% species coverage for planning units without abundance weighting (*q* = 0), following recommendations of Chao, Gotelli, et al. (2014) and Hsieh et al. (2016). In this approach, sampling theory links rarefaction and extrapolation to allow diversity comparison across sites of varied sampling intensity critical in a megadiverse undersampled landscape (Cazzolla Gatti et al., 2022; Trethowan, Brambach, et al., 2024). Extrapolation was repeated for trait alpha diversity, which resulted in a sum of the number of functional entities that exceeded the threshold of the mean distance between any 2 individuals randomly selected from the pooled assemblage (Chao, Chiu, et al., 2014). The trait distances were Gower distances, with each flower color or habit downweighted so that the total weights for flower colors and habits were equal to continuous trait variables. The equation to calculate alpha Hill diversity was as follows (Chao, Chiu, et al., 2014):

(2)

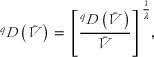

where *q* is the abundance weighting parameter, $\bar{V}$ is abundance weight, D is the type of diversity in question (e.g., species or trait), and λ is species (equal to 1) or trait diversity (equal to 2).

To aid complementarity during prioritization, we used beta diversity layers (Mokany et al., 2011; Socolar et al., 2016). We used the Hill metric not weighted by abundance (*q* = 0) Jaccard‐type similarity to measure effective proportion of the pooled assemblage that is shared with each planning unit (Chao, Chiu, et al., 2014; Li, 2018). Jaccard‐type similarities were averaged for each planning unit and then subtracted from one to give measures of dissimilarity per planning unit. This was repeated for trait beta diversity while retaining the threshold distance between functional entities as the mean Gower trait distance between any 2 individuals randomly selected from the pooled assemblage (Chao et al., 2019; Magneville et al., 2022). Following recommendations of Chao et al. (2019), we weighted this metric by estimated abundance (*q* = 2) (Chao et al., 2019). Equations used to derive Jaccard‐type similarity Hill beta diversity are as follows (Chao, Chiu, et al., 2014):

(3) $$U_{qN}^{*}\left(\bar{V}\right)=\frac{{\left[1{/}^{q}AD_{\beta }\left(\bar{V}\right)\right]}^{1-q}-{\left(1/N\right)}^{\lambda \left(1-q\right)}}{1-{\left(1/N\right)}^{\lambda \left(1-q\right)}},$$

where variables are the same as for Equation (2) and N is the number of assemblages per planning unit. We calculated the corresponding dissimilarity measure with the following equations:

(4) $$1-U_{qN}^{*}\left(\bar{V}\right),$$

where $AD_{\beta }$ is the ratio of alpha ($D_{\alpha }$) and gamma diversity ($D_{\gamma }$), and

(5)

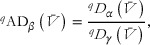

where $D_{\gamma }$ is the mean value in the pooled assemblage of species or functional entities.

Our cost layer was the mean score of scaled (0–1) accessibility or the inverse travel time to a planning unit (Nelson et al., 2019), human footprint (Venter et al., 2016), and human population density in 2020 (CIESIN, 2018). We used this to represent the assumption that land is of greater value closer to population hubs where wages and demand are higher; we did not use future deforestation estimates, as in previous work (Parsch et al., 2025), because we aimed to determine how conservation plans could limit deforestation expansion.

To improve connectivity of prioritized areas, we added a 0.003 penalty to increase importance of selecting planning units that are spatially clumped together and an edge factor of 0.5 to limit penalization of units on coastlines; these values were selected because they provide relevant practical scenarios for stakeholders and decision makers (Beger et al., 2022; Beyer et al., 2016; Hanson et al., 2025). We carried out prioritization for 2 scenarios, one for 30% land area following CBD targets and another for 70% land area following the Manokwari Declaration (Cámara‐Leret, Schuiteman, et al., 2019; Parsch et al., 2022). The 30% scenario had a budget of 20% of total land cost and the 70% scenario used a 35% budget. We selected these budgets to provide realistic scenarios given the budget limits of local government (Cámara‐Leret, Schuiteman, et al., 2019). Within these budget constraints, we aimed to select the scenario with minimum shortfall for the objective to conserve the target percentage for all features (Arponen et al., 2005). We also repeated the prioritization with current protected areas locked into the designations (Appendix S5); however, our analyses of trait contribution to prioritization were not based on these designations because we required results to be informed primarily by botanical diversity.

### Trait diversity impact on prioritization

To measure the impact of trait diversity metrics on the prioritization, we first calculated the importance values of planning units for each diversity feature. Importance values are the likelihood that a given unit is needed to achieve a specified set of conservation targets or how achieving these targets is reduced if the unit is not protected (Ferrier et al., 2000; Pressey et al., 1994). Specifically, these values are the proportion of the total number of prioritization scenarios that fulfil the conservation target that includes the planning unit in question but does not fulfil the targets if that unit is removed (Ferrier et al., 2000). This can be expressed as

(6) $$\mathrm{importanc}e_{\mathrm{pu}}=\frac{\mathrm{scenario}s_{\mathrm{pu}\;\mathrm{included}}-\mathrm{scenario}s_{\mathrm{pu}\;\mathrm{removed}}}{\mathrm{scenario}s_{\mathrm{pu}\;\mathrm{included}}+\mathrm{scenario}s_{\mathrm{pu}\;\mathrm{excluded}}},$$

where $\mathrm{scenario}s_{\mathrm{pu}\;\mathrm{included}}$ is the number of prioritization scenarios that achieve the conservation target and include the planning unit pu in question, $\mathrm{scenario}s_{\mathrm{pu}\;\mathrm{removed}}$ is the number of prioritization scenarios that do not achieve the conservation target if that planning unit is removed, and $\mathrm{scenario}s_{\mathrm{pu}\;\mathrm{excluded}}$ is the number of scenarios that achieve the conservation target but do not include the planning unit in question. This was repeated using each of our diversity features separately as the conservation target and yielded 4 separate $\mathrm{importanc}e_{\mathrm{pu}}$ values for prioritization of species and trait alpha and beta diversity.

We calculated trait diversity impact as $\mathrm{importanc}e_{\mathrm{pu}}$ for trait alpha diversity minus the $\mathrm{importanc}e_{\mathrm{pu}}$ for species alpha diversity and then repeated this calculation for beta diversity (Kujala et al., 2018). Positive scores showed units more critical for conservation of trait diversity, and negative values showed units more fit for conservation of species diversity. Trait priority units were those with trait diversity impact >0 for alpha or beta diversity. Planning units with both alpha and beta trait impact scores below zero were species priority units.

### Trait diversity and prioritization of carbon stocks and high deforestation risk areas

We repeated the prioritizations with aboveground carbon stocks and deforestation probability by 2050 as additional features to guide conservation decisions. Aboveground carbon stock estimation was derived from a Global Ecosystem Demography model underpinned by 2 NASA spaceborne lidar datasets, Global Ecosystem Dynamics Investigation and ICE, Cloud, and Land Elevation Satellite 2 (Ma et al., 2023). Deforestation probability was based on an Indonesia‐wide dataset that expanded the spatial scale of previous deforestation prediction exercises (Seaman et al., 2025; Voigt et al., 2022, 2021). Future deforestation risk predictions were produced using a spatially explicit modeling framework developed by Rosa et al. (2013). The model was trained on data of past forest loss from 1990 to 2015 and calibrated on forest lost from 2016 to 2020 (Vancutsem et al., 2021). The model dynamically updates future deforestation predictions in 5‐year time steps and explicitly accounts for uncertainty and the contagious and stochastic characteristic of deforestation. For each time step, the model is run for 100 interactions and summed, and the final product is a summed probability of deforestation for each pixel. We then compared $\mathrm{importanc}e_{\mathrm{pu}}$ scores for carbon stocks and deforestation risk for those units designated as of priority for trait or species conservation.

## RESULTS

The addition of species’ traits improved model predictions of species occurrence (δ RMSE average across models = −0.24) (Figure 2c). Among all traits, leaf length provided the greatest model improvement (δ RMSE = −0.65). The next best traits for model improvement were red (δ RMSE = −0.58) and purple (δ RMSE = −0.55) flowers. Brown flowers did not improve models (δ RMSE = 0.01). Likewise, habit traits did not provide great model improvement (Figure 2c). Whether or not species are herbs gave the best improvement among habit data (δ RMSE = −0.5), whereas climbing habit yielded the least improvement (δ RMSE = 0.09). Size traits varied in their ability to improve the model. Inclusion of leaf width provided improvement (δ RMSE = −0.2) as did plant size (δ RMSE = −0.27). Reproductive size traits, however, offered less improvement (fruit size δ RMSE = 0.03; flower size δ RMSE = −0.03).

There was variation in CPO change with trait addition to models (e.g., across all species δ CPO SD = 0.0065), as expected with thousands of species–site combinations. Median δ CPO values suggested traits boosted predictions for species with fewer occurrence records (Figure 2d). The 2 rarest species quartiles showed the greatest model improvement and increases in CPO with the inclusion of traits (Figure 2d). This was not consistent across traits. Only across flower colors did the model consistently improve for the rarest species (Figure 2d). Leaf and plant size similarly improved occurrence models for rarer species, but fruit and flower size and habit categories did not (Figure 2d). The exception was tree versus nontree species models, which had greater CPO values for rarer quartiles (Figure 2d).

**FIGURE 3:**
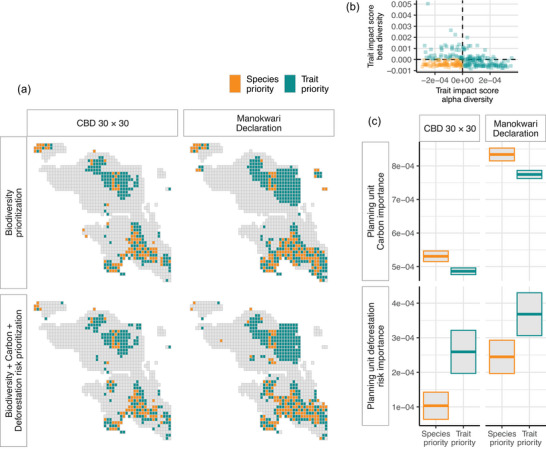
(a) Distribution of priority areas for conservation based on plant trait and plant species diversity across the Bird's Head Peninsula, New Guinea, relative to planning units for the Convention on Biological Diversity (CBD) 30% and the Manokwari Declaration 70% protection targets and for prioritization of conservation areas based on biodiversity data (species and trait alpha and beta diversity) only and based on biodiversity data and carbon stock and deforestation risk features, (b) alpha and beta diversity trait impact scores for species and trait priority planning units, and (c) importance scores for carbon stock and 2050 deforestation risk for trait and species priority planning units (central line in bars, mean; bar ends, SD).

Overall, trait diversity contributed to the selection of land for protection more than species diversity alone (Figure 3a). The scenario for 30% biodiversity protection had 185 trait priority units and 89 species priority units. Thirty percent coverage for biodiversity, carbon, and deforestation risk was similar: 188 trait priority units and 77 species priority units. With 70% coverage for just biodiversity, there were 283 trait priority units compared with 120 species priority units. Seventy percent coverage for biodiversity, carbon, and deforestation risk was again similar, with 283 trait priority units and 117 species priority units (Figure 3a).

Trait priority units had a trait impact score >0 for alpha or beta diversity (Figure 3b). For 30% and 70% coverage prioritization scenarios, more planning units had a trait impact score >0 (i.e., prioritized more for trait than species diversity) for alpha diversity (30% coverage proportion of sites 0.5; 70% coverage 0.47) than for beta diversity (30% coverage 0.32; 70% coverage 0.36). A smaller proportion of planning units had positive trait impact scores for both alpha and beta diversity (30% coverage 0.14; 70% coverage 0.13). This was replicated for the prioritization of biodiversity, carbon, and deforestation risk (trait alpha priority 30% coverage proportion of sites 0.55; 70% coverage 0.47; trait beta priority 30% coverage 0.31; 70% coverage 0.37; both trait alpha and beta priority 30% coverage 0.15; 70% coverage 0.13).

Trait priority units had greater importance for deforestation risk (30% coverage, mean [SD] = 0.00026 [0.00085] importance score; 70% coverage, mean = 0.00037 [0.001]) than species priority units (30% coverage, mean = 0.00011 [0.00035]; 70% coverage, mean = 0.00024 [0.00052]) (Figure 3c). Whereas, species priority units had greater importance for carbon stock prioritization (30% coverage, mean = 0.00053 [0.00014]; 70% coverage, mean = 0.00083 [0.00021]) than trait priority units (30% coverage, mean = 0.00049 [0.00014]; 70% coverage, mean = 0.00077 [0.0002]) (Figure 3c).

**FIGURE 4:**
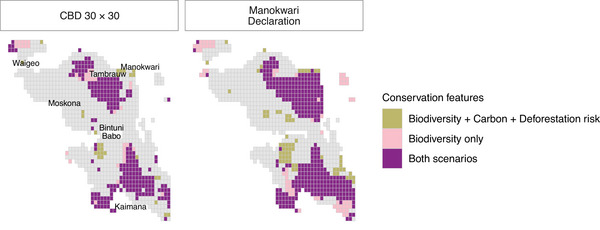
Bird's Head Peninsula, New Guinea, planning units prioritized for conservation selected using biodiversity data (plant species and plant trait alpha and beta diversity) and other features (added carbon stock and deforestation risk) to meet the Convention on Biological Diversity (CBD) 30% and the Manokwari Declaration 70% protection targets.

Large areas were consistently in the Tambrauw Mountains and the Bird's Neck region, including Kaimana (Figure 4). Key lowland areas in Waigeo and around Bintuni and Moskona were also selected (Figure 4). The prioritizations for biodiversity, carbon stock, and deforestation risk additionally selected the lowlands around Babo and the remaining forest around Manokwari (Figure 4). For CBD 30 × 30 scenarios, 38 units were selected only in the biodiversity scenario, 29 units were selected only in the biodiversity, carbon stock, and deforestation risk scenario, and 237 units were selected in both scenarios. For 70% protection, 67 units were selected only in the biodiversity scenario, 64 units were selected only in the biodiversity, carbon stock, and deforestation risk scenario, and 338 units were selected in both scenarios (Figure 4).

## DISCUSSION

Our results add to the evidence base that species’ traits are another dimension of diversity that can be used to aid conservation planning (Butt & Gallagher, 2018; Gallagher et al., 2021; Santini et al., 2016). We found that traits improved performance of joint species distribution models and showed how trait diversity can be incorporated into conservation planning in species‐rich landscapes. We provided a biodiversity‐based spatial plan for the Bird's Head Peninsula West Papua that was guided by local government conservation policy to protect 70% of one of the planet's most diverse ecosystems.

Trait data held in taxonomic descriptions have a long history of use in evolutionary studies and are beginning to aid functional macroecology (Graham et al., 2004; Velásquez‐Puentes et al., 2023). Measurements based on historic collections that encompass species concepts, published as descriptions in taxonomic revisions and floras, replicate data often collected in the field by functional ecologists but at a greater phylogenetic scale. Descriptions also include reproductive traits that are key aspects of plant function but are difficult to measure in the field due to the irregular flowering of wet tropical species (E‐Vojtkó et al., 2020). We showed how data derived from historic collections can fill trait data gaps for megadiverse floras that in turn can be used to benefit evidence‐led conservation strategy.

Critically, we showed that by embracing trait data, limited data can be mitigated and enhanced for the thousands of tropical species. Models of species occurrence have been critical for species‐level conservation planning (Franklin, 2013). For instance, on other islands in tropical Asia, stacked mammal species occurrence models in Borneo and Sumatra were used to assess the extent and connectivity of protected areas (Pinondang et al., 2024; Sibarani et al., 2019; Struebig et al., 2024, 2015). Phylogenetic relationships and trait–environment interactions allowed us to model species occurrence jointly for New Guinea's tropical flora. The benefits of this extra information are doubly important in the megadiverse tropics, where occurrence data are often insufficient to build single‐species distribution models for the thousands of species that are naturally rare (Enquist et al., 2019; Feeley & Silman, 2011; Zhang et al., 2020). Conservation planning based on rare species and their protection should particularly benefit from the addition of trait data (Dinerstein et al., 2024).

Our occurrence predictions did not improve consistently with the use of data across the spectrum of plant size and reproduction form and function. Size traits were variable in their positive effects on predictions. Leaf length measures consistently improved predictions of species occurrence, more so than plant size despite its being a key metric for determining ecosystem function (e.g., Cadotte, 2017). Similarly, habit data did relatively little to improve models, whereas leaf size better predicted species presence across the Bird's Head environmental gradient (Sterck et al., 2006).

Flower and fruit size also did not provide major predictive improvement, perhaps due to the competing effects of greater productivity in the lowlands versus large pollinators at higher elevations, where vertebrate diversity peaks alongside dominant, large dispersers, such as hornbills, cassowaries, and fruit bats present across elevations (Tallowin et al., 2017). Flower colors, except brown, provided good model improvement. Flower color effect on species distribution is controlled by the links between climate, pigment production, and pollinator diversity; if all those constraints are encapsulated by use of flower color in distribution models, it will likely be beneficial for landscapes beyond New Guinea (Dalrymple et al., 2020; Trethowan, Jennings, et al., 2024). The Bird's Head Peninsula spans the world's largest mangrove complex (e.g., Sasmito et al., 2020; Sillanpää et al., 2024), lowland wet forest, and mountain peaks that are rich in endemic species (Gibbs, 1917; Jennings et al., 2023). These landscapes often contain rare species, and models parameterized with flower colors and leaf size improved model results for rare species most of all. These traits are likely tied closely to environments and habitats that contain these rare species; for instance, a gradient of white flower diversity peaks in the lowlands, and there are likely more colorful flower species at higher elevations (Delmas et al., 2020; Trethowan, Jennings, et al., 2024). Similarly, the endemic rich shrublands and mangroves often host species with small leaves that limit transpiration water loss due to greater exposure to sun and wind in the former and salt in the latter (Wei et al., 2025; Zorger et al., 2025). Traits therefore aid the prediction of species occurrence, although traits may differ in whether they improve predictions for rare or common species.

Our results showed that across the landscape, single contiguous conservation areas can be selected to preserve both species and trait diversity. No large area selected was a result of just high species or trait diversity values. The large areas selected to achieve conservation goals were not uniform in their patterns of trait or species priorities. In northern Bird's Head, large areas were selected as either trait or species priority, whereas in the south priority units were more interspersed with both trait and species priority units. These results may shift with greater occurrence sampling particularly in currently uncollected areas; despite this, the framework appeared to capture complexity in regional diversity distribution and to construct a coherent conservation plan.

Trait diversity is a good predictor of ecosystem resilience because traits determine whether species can tolerate novel stressors or whether a system can return to equilibrium postdisturbance (de Bello et al., 2021). Trait‐diversity‐focused prioritization of conservation areas should therefore help initiatives build more resilient future ecosystems (Gladstone‐Gallagher et al., 2019; Ross et al., 2021). Traits can improve predictions of susceptibility to changing climates (Andrew et al., 2022; Comte et al., 2024), and in situ conservation actions can be influenced by trait information (e.g., cycad species conservation in Mexico [Álvarez‐Yépiz et al., 2019]). Trait diversity has been incorporated in spatial plans for multiple taxa, including freshwater fish, crocodiles, mammals, and parrots (Brum et al., 2017; Griffith et al., 2023; Kosman et al., 2019; Strecker et al., 2011). For plants, belowground traits, because of their close relationship with soil water and nutrient availability, are a key parameter of ecosystem resilience, and their integration into conservation prioritization in the tropics would be valuable (Kühn et al., 2023; Laughlin et al., 2021). Future work is required that incorporates more species and traits across larger areas and uses counterfactual analyses to test resilience of different potential prioritizations under future climatic and anthropogenic stressors (Bicknell et al., 2023; Ortiz‐Ross & Blumstein, 2024). These plans will similarly need to consider all dimensions of diversity (functional, trait, species, and phylogenetic) to build in‐depth spatial conservation strategies (Cadotte & Tucker, 2018).

There are a number of limitations to our study. We had relatively sparse data for species’ traits and occurrences. To address this, we had to use a large number of herbarium records in conjunction with taxonomic literature, particularly for little known species and in undersampled lowlands. Our work therefore exemplifies the direct links between collections, mainly a result of taxonomic study, and conservation planning. For our co‐occurrence modeling framework, we relied on the assumption that in 11 × 11‐km planning units species interact; however, at this scale all species are unlikely to depend on each other. However, this approach is not subject to the erroneous assumption that all species do not interact, which is the assumption when stacking single‐species distribution models (Poggiato et al., 2021). Our results should be understood in the context of the relatively poor performance of the co‐occurrence models (Appendix S4), although model improvement as the result of trait parameter addition is clear. Additionally, work ahead of formal decision‐making could add more depth by identifying the sensitivity of prioritizations to varying scenarios of land cost, budgets, and connectivity parameters (Margules & Pressey, 2000).

We produced a biodiversity‐focused plan that could limit habitat loss and retain forest carbon stocks. Prioritizations highlighted the importance of the upland Tambrauw–Arfak massif and indicated that lower elevations around Moskona, Bintuni, Babo, Kaimana, Manokwari, and Waigeo are potentially key conservation areas (Figures 1 & 4). This selection of lowland areas occurred despite the lack of data on belowground carbon stocks, which can greatly exceed aboveground carbon stocks in mangroves and peat swamp forests. Our results and future work integrating peat swamp data will make the case for greater lowland forest conservation, which is lacking in the current protected area network (Parsch et al., 2022; Sloan et al., 2019).

We used species’ traits from taxonomic literature to aid conservation prioritization in the megadiverse tropics. Given the dearth of data available for tropical plant species, historic collections, as a multidimensional data source, should be embraced when building the evidence basis for conservation decisions. The trait data specimens offer, and resulting descriptions, allowed us to improve models of the distribution of rare species, where the underlying distribution data again came from herbarium specimens. We incorporated trait diversity into spatial plans for hundreds of species, which by embracing historic collections and resulting literature is scalable to thousands of plant species. Modern conservation methods and action can therefore benefit from collections held in museums and herbaria, particularly in the tropics, where data are sparse for most organisms across the evolutionary tree of life.

## Supporting information



Supplementary Material

Supplementary Material

Supplementary Material

Supplementary Material

Supplementary Material

## Data Availability

Data and Rmarkdown to reproduce the study are available from https://zenodo.org/records/17531025.
