# Supplementary material for: Informing spatial conservation prioritization with species’ traits
Source: Conserv Biol. 2025 Dec 13;40(2):e70199. doi: 10.1111/cobi.70199 (PMC13036316; doi:10.1111/cobi.70199)
Supplement: Supplementary file 1 — Supplementary Material [file COBI-40-e70199-s002.docx]

Appendix S1. Above: Number of occurrences within planning units across five distribution datasets used. Below: log transformed number of occurrences for species across five distribution datasets, split into rarity quartiles shown by color.

Appendix S2. Mean and standard deviation occurrence probability across distribution datasets used. Values scaled for each dataset for better visual clarity of results.

Appendix S3. Root Mean Square Error (RMSE) for 10-fold validation of trait parameterized co-occurrence models across five presence-background datasets.

| Trait | **training_boundbox** N = 10*^1^* | **training_buffer150** N = 10*^1^* | **training_buffer50** N = 10*^1^* | **training_elevation** N = 10*^1^* | **training_specimen** N = 10*^1^* | **validation_boundbox** N = 10*^1^* | **validation_buffer150** N = 10*^1^* | **validation_buffer50** N = 10*^1^* | **validation_elevation** N = 10*^1^* | **validation_specimen** N = 10*^1^* |
| --- | --- | --- | --- | --- | --- | --- | --- | --- | --- | --- |
| Size | -0.811 (0.022) | -0.856 (0.009) | -0.692 (0.496) | -0.805 (0.004) | 0.005 (0.910) | 0.422 (0.180) | 0.812 (0.100) | 0.734 (0.380) | 0.368 (0.021) | 0.005 (0.914) |
| Leaf length | -0.804 (0.021) | -0.804 (0.021) | -0.806 (0.023) | -0.816 (0.008) | 0.022 (0.899) | 0.394 (0.192) | 0.401 (0.187) | 0.357 (0.081) | 0.435 (0.050) | 0.019 (0.921) |
| Leaf width | -0.822 (0.024) | -0.851 (0.014) | -0.686 (0.450) | -0.825 (0.009) | 0.006 (0.909) | 0.482 (0.168) | 0.775 (0.148) | 0.771 (0.341) | 0.530 (0.147) | 0.001 (0.907) |
| Shrub | -0.861 (0.010) | -0.833 (0.064) | -0.443 (0.613) | -0.864 (0.001) | 0.026 (0.897) | 0.833 (0.109) | 0.908 (0.166) | 0.524 (0.265) | 0.870 (0.021) | 0.027 (0.931) |
| Climbing | -0.864 (0.005) | -0.864 (0.002) | -0.573 (0.467) | -0.864 (0.000) | 0.004 (0.910) | 0.846 (0.055) | 0.860 (0.019) | 0.653 (0.385) | 0.868 (0.010) | 0.002 (0.912) |
| Epiphyte | -0.838 (0.021) | -0.836 (0.018) | -0.622 (0.410) | -0.850 (0.005) | 0.002 (0.912) | 0.588 (0.140) | 0.584 (0.129) | 0.513 (0.196) | 0.762 (0.074) | 0.004 (0.917) |
| Herb | -0.821 (0.022) | -0.819 (0.022) | -0.831 (0.067) | -0.848 (0.007) | 0.001 (0.912) | 0.486 (0.156) | 0.482 (0.153) | 0.461 (0.198) | 0.655 (0.082) | -0.002 (0.911) |
| Tree | -0.859 (0.009) | -0.858 (0.008) | -0.504 (0.588) | -0.856 (0.002) | 0.003 (0.911) | 0.859 (0.031) | 0.868 (0.031) | 0.557 (0.353) | 0.880 (0.022) | 0.002 (0.915) |
| Brown | -0.492 (0.722) | -0.864 (0.002) | -0.566 (0.509) | -0.866 (0.000) | 0.174 (0.894) | 0.462 (0.671) | 0.871 (0.027) | 0.627 (0.351) | 0.865 (0.003) | -0.173 (0.895) |
| Orange | -0.812 (0.037) | -0.821 (0.022) | -0.811 (0.044) | -0.849 (0.007) | 0.173 (0.894) | 0.457 (0.209) | 0.489 (0.151) | 0.469 (0.183) | 0.679 (0.083) | -0.173 (0.895) |
| Pink | -0.817 (0.056) | -0.829 (0.019) | -0.638 (0.349) | -0.847 (0.006) | 0.176 (0.895) | 0.526 (0.277) | 0.576 (0.139) | 0.658 (0.377) | 0.675 (0.084) | -0.172 (0.896) |
| Purple | -0.810 (0.032) | -0.817 (0.020) | -0.806 (0.036) | -0.844 (0.010) | 0.173 (0.894) | 0.431 (0.192) | 0.459 (0.144) | 0.417 (0.118) | 0.619 (0.105) | -0.173 (0.894) |
| Red | -0.808 (0.030) | -0.814 (0.021) | -0.815 (0.045) | -0.835 (0.013) | 0.174 (0.894) | 0.418 (0.191) | 0.437 (0.148) | 0.416 (0.146) | 0.562 (0.125) | -0.174 (0.894) |
| White | -0.795 (0.109) | -0.841 (0.019) | -0.733 (0.298) | -0.849 (0.009) | 0.174 (0.895) | 0.480 (0.389) | 0.672 (0.160) | 0.642 (0.296) | 0.702 (0.106) | -0.172 (0.893) |
| Yellow | -0.634 (0.540) | -0.857 (0.009) | -0.459 (0.581) | -0.846 (0.008) | 0.173 (0.894) | 0.430 (0.683) | 0.753 (0.101) | 0.626 (0.393) | 0.618 (0.092) | -0.173 (0.895) |
| Flower size | -0.639 (0.546) | -0.865 (0.001) | -0.464 (0.585) | -0.864 (0.001) | 0.174 (0.894) | 0.449 (0.685) | 0.846 (0.034) | 0.641 (0.397) | 0.819 (0.043) | -0.174 (0.894) |
| Fruit size | -0.502 (0.729) | -0.858 (0.003) | -0.458 (0.577) | -0.861 (0.001) | 0.175 (0.894) | 0.418 (0.784) | 0.834 (0.080) | 0.659 (0.404) | 0.874 (0.017) | -0.172 (0.896) |
| *^1^*Mean (SD)  *Appendix S4.* Mean and standard deviation Root Mean Square Error (RMSE) for 10-fold validation of trait parameterised co-occurrence models across five presence-background datasets. | | | | | | | | | | |

Appendix S5. Bird’s Head Peninsula prioritized planning units selected using biodiversity data (species and trait alpha and beta diversity) and/or those selected with added carbon stock and deforestation risk features. Results shown for the CBD 30 % and the Manokwari Declaration 70 % protection targets. These results have current designated protected areas locked into the prioritization.
