# Supplementary figures and images for: Informing spatial conservation prioritization with species’ traits
Source: Conserv Biol. 2025 Dec 13;40(2):e70199. doi: 10.1111/cobi.70199 (PMC13036316; doi:10.1111/cobi.70199)

—●— training    -▲- validation

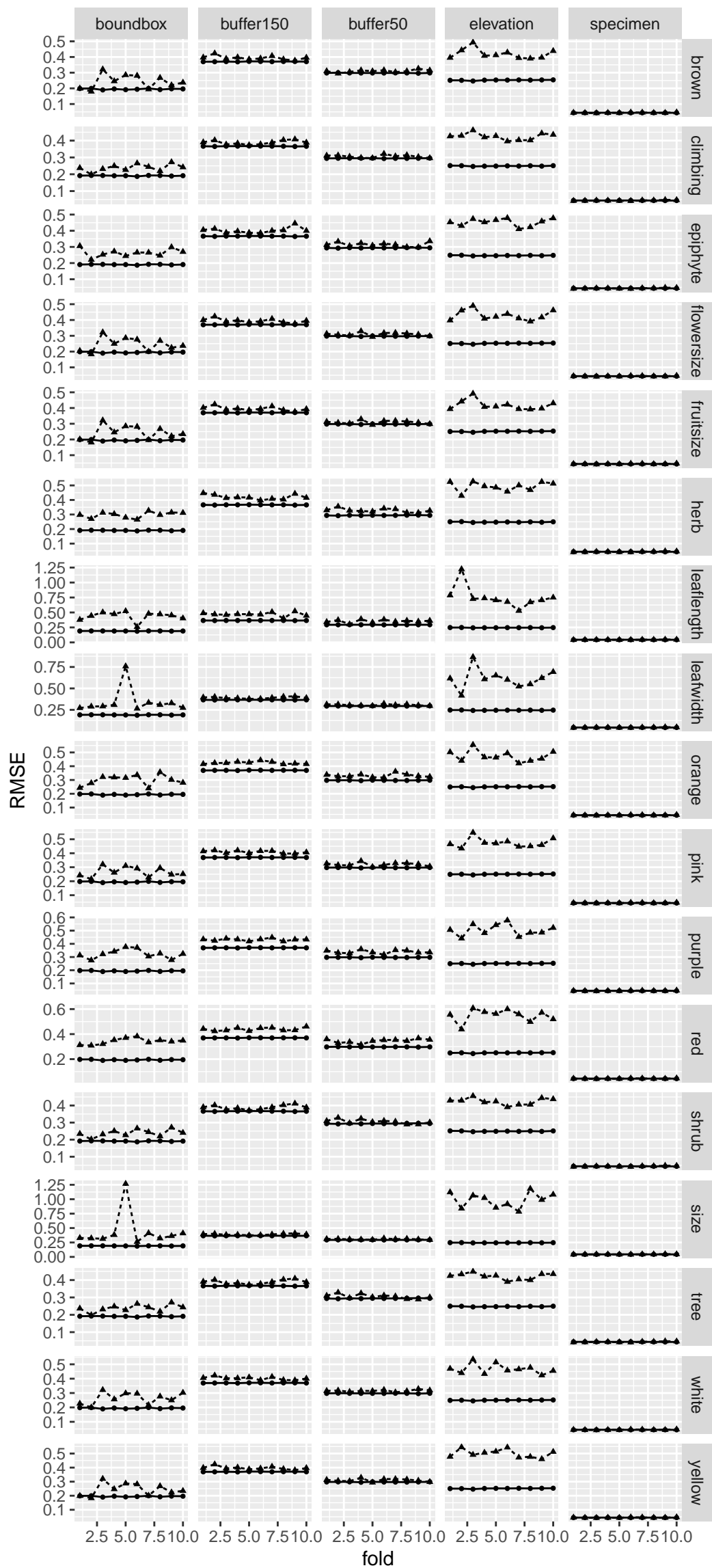

Supplement: Supplementary file 2 — Supplementary Material [file COBI-40-e70199-s004.pdf]
