# Supplementary material for: Informing spatial conservation prioritization with species’ traits
Source: Conserv Biol. 2025 Dec 13;40(2):e70199. doi: 10.1111/cobi.70199 (PMC13036316; doi:10.1111/cobi.70199)
Supplement: Supplementary file 3 — Supplementary Material [file COBI-40-e70199-s001.pdf]

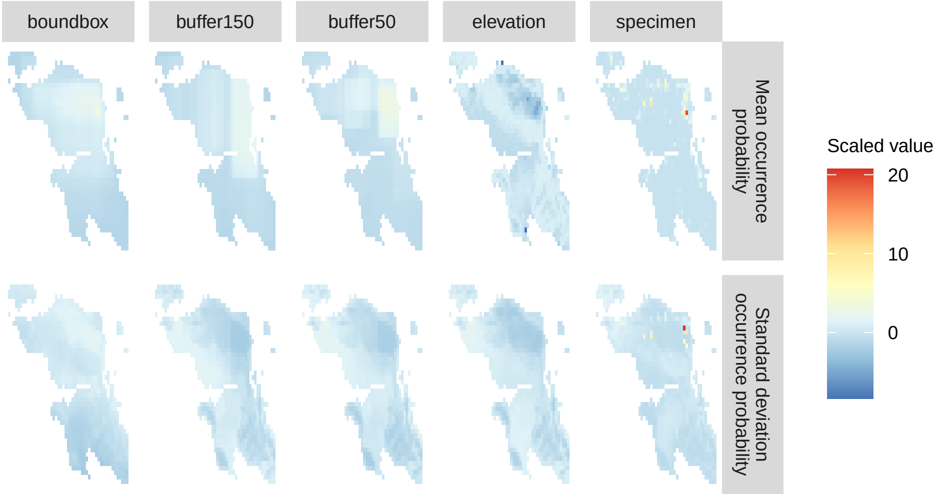
boundbox
buffer150
buffer50
elevation
specimen
Mean occurrence probability
Standard deviation occurrence probability
Scaled value
20
10
0
