# Supplementary material for: Informing spatial conservation prioritization with species’ traits
Source: Conserv Biol. 2025 Dec 13;40(2):e70199. doi: 10.1111/cobi.70199 (PMC13036316; doi:10.1111/cobi.70199)
Supplement: Supplementary file 4 — Supplementary Material [file COBI-40-e70199-s003.pdf]

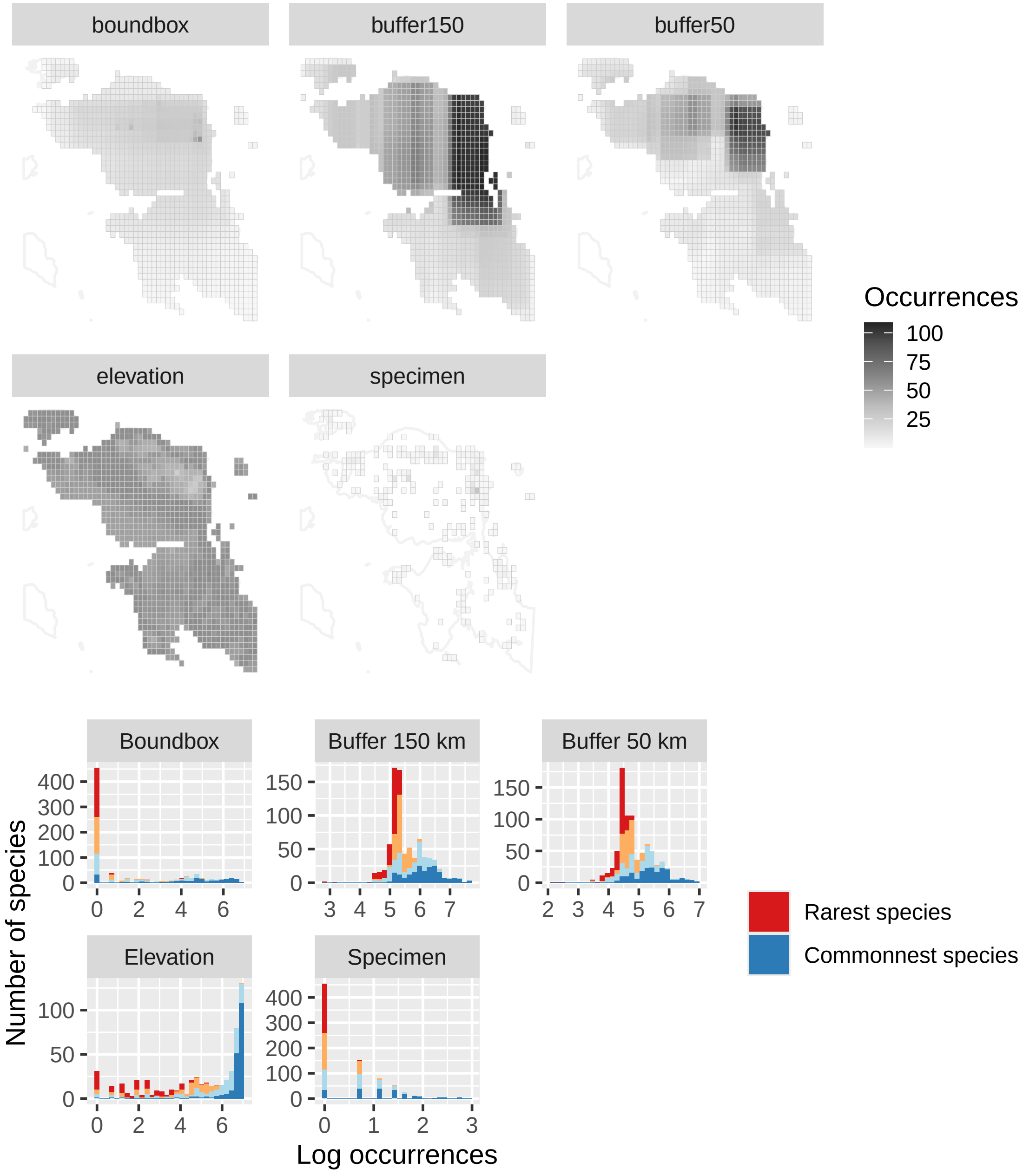
boundbox
buffer150
buffer50
elevation
specimen
Occurrences
100
75
50
25
Boundbox
Buffer 150 km
Buffer 50 km
Elevation
Specimen
Number of species
Log occurrences
Rarest species
Commonnest species
