# Supplementary material for: Informing spatial conservation prioritization with species’ traits
Source: Conserv Biol. 2025 Dec 13;40(2):e70199. doi: 10.1111/cobi.70199 (PMC13036316; doi:10.1111/cobi.70199)
Supplement: Supplementary file 5 — Supplementary Material [file COBI-40-e70199-s005.pdf]

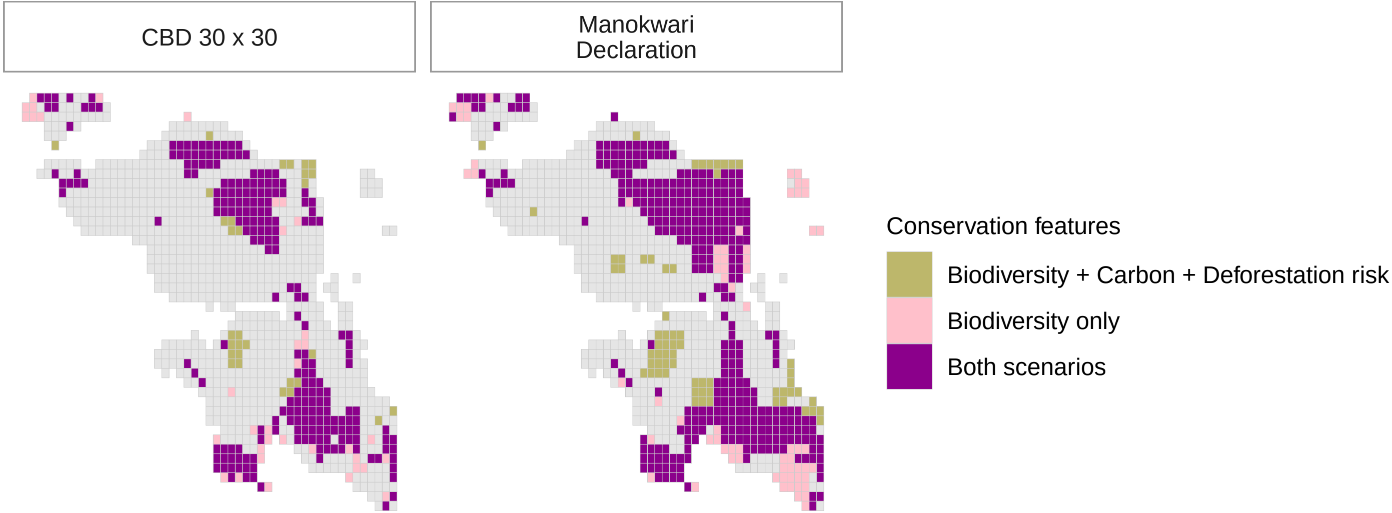
CBD 30 x 30
Manokwari Declaration
Conservation features
Biodiversity + Carbon + Deforestation risk
Biodiversity only
Both scenarios
